# A Modern Overview of the Potential Therapeutic Effects of Psilocybin in the Treatment of Depressive Disorders, Treatment-Resistant Depression, and End-of-Life Distress

**DOI:** 10.7759/cureus.80707

**Published:** 2025-03-17

**Authors:** Fejzic Dino

**Affiliations:** 1 Department of Regulatory Affairs and Development, Bosnalijek JSC, Sarajevo, BIH

**Keywords:** depression, end-of-life distress, hallucinogens, psilocybin, psychedelics, treatment resistant depression

## Abstract

The purpose of this review is to provide a comprehensive overview of the current findings and data on the therapeutic effects of psilocybin, a naturally occurring psychedelic alkaloid primarily found in *Psilocybe* mushrooms. This review covers psilocybin’s efficacy and safety profile, therapeutic effects, proposed indications and contraindications, drug-drug interactions, adverse reactions, pharmacokinetics, pharmacodynamics, and dosing regimens as treatment guidelines. The goal is to offer a consolidated resource containing the essential pharmaceutical information on psilocybin currently available.

## Introduction and background

Depression is one of the most prevalent illnesses of the modern era and remains a leading cause of disability worldwide [1]. Estimates suggest that approximately 300 million people globally suffer from major depressive disorder (MDD) [2,3]. Despite its widespread impact, depression is still poorly understood.

Each year, over 700,000 people die by suicide, a number expected to rise given current global challenges [4]. While not all individuals who die by suicide have depression, psychological studies have identified depression as the most common illness among suicide victims [5]. Suicide is the most alarming complication of depression, with the pooled lifetime prevalence of suicide attempts among patients estimated to be between 27% and 34%, nearly 20 times higher than in the general population [6]. It is important to note that suicide is not always directly caused by depression, nor is every suicide attempt an intention to end one’s life; often, it represents a desperate plea for help. Tragically, suicide is also the second leading cause of death among young people aged 15-29 [7].

Although depression is classified as a psychiatric illness and is one of the most common alongside anxiety disorders, it has a substantial biological component, largely resulting from biochemical changes in the CNS. Unfortunately, its exact etiology remains unknown [6].

Depression develops through a complex interplay of environmental, societal, and biological factors. Individuals living in impoverished regions, war zones, or areas marked by conflict and resource scarcity face a higher risk of developing depression. Environmental stressors, such as instability and lack of access to basic needs, significantly contribute to mental health challenges. Genetic predispositions also play a critical role, highlighting the complex relationship between nature and nurture in mental health [8].

Currently, there are no chemical markers or laboratory tests to diagnose depression; psychiatric evaluation remains the only viable diagnostic approach. However, recent studies have identified potential biomarkers for depression. One proposed biomarker is brain-derived neurotrophic factor (BDNF), a molecule essential for neurogenesis and neuroplasticity in the hippocampus. Depressed individuals often exhibit reduced BDNF expression in the brain’s limbic areas due to neuronal atrophy. Serotonin and its receptors help regulate BDNF levels, and prolonged treatment with selective serotonin reuptake inhibitors (SSRIs) has been shown to increase BDNF levels in humans [1].

Other potential biomarkers include IL-6, IL-12, and CRP, as patients with MDD tend to have elevated levels of pro-inflammatory markers compared to healthy individuals. Treatment-resistant depression (TRD) is also associated with heightened pro-inflammatory markers. Peripheral inflammation allows cytokines and immune cells to cross the blood-brain barrier, potentially altering the kynurenine pathway and affecting tryptophan availability [1]. Additionally, a decreased ratio of 5-hydroxyindoleacetic acid to serotonin (5-HT) in the cerebrospinal fluid has been observed in depressed patients, indicating reduced serotonergic neurotransmission. Long-term antidepressant use helps normalize these levels alongside symptom reduction [9].

These biomarkers are currently theoretical and not yet applied to diagnose depression. However, identifying reliable markers could greatly improve diagnostic accuracy and enhance the ability to monitor recovery with standardized assessments.

Depression is generally poorly treated, with most patients not receiving optimal care [10]. Given that depression has both biological and psychiatric aspects, effective treatment requires addressing both dimensions. However, most patients are simply prescribed antidepressants with little to no psychological support, largely due to a shortage of specialists [10]. Ideally, treating depressive disorders should involve psychotherapy or a combination of psychotherapy and medication, rather than medication alone [11,12].

Studies estimate that only 30-40% of patients diagnosed with depression, particularly MDD, achieve full remission with first-line treatment, often necessitating adjustments when initial treatments are ineffective. Unfortunately, around one-third of patients do not respond even after trying four different antidepressants [6]. While there is no universal definition, this condition is commonly referred to as TRD, typically described as depression unresponsive to at least two antidepressants with different mechanisms of action [1].

First-line treatments usually involve SSRIs, but these drugs have significant limitations. They often require weeks to produce noticeable effects, many patients experience side effects, and their efficacy is limited, with 30-50% of patients not responding to treatment [3,13-15]. The possibility exists that increased extracellular serotonin levels from SSRI use activate not only 5-HT receptor subtypes that alleviate depression but also 5-HT1A autoreceptors and 5-HT2A/C receptors, which may exacerbate depressive symptoms [16].

Given the prevalence of depression and the relatively low efficacy of current antidepressants, there is an urgent need for innovative treatments that provide higher efficacy and faster onset than standard therapies [17]. It is particularly crucial to develop drugs that produce immediate effects, especially for patients with suicidal tendencies and those facing terminal illnesses.

Psychedelic substances are emerging as promising alternatives for treating mood disorders. Compounds like psilocybin and lysergic acid diethylamide (LSD) have a long history of use, and research into their medical applications is gaining momentum despite legal barriers. Psilocybin, a naturally occurring compound found in certain mushroom species, has been used historically in various religious and ceremonial practices. More recently, psilocybin has attracted substantial interest from the scientific community for its potential therapeutic effects in treating depressive spectrum disorders [18].

## Review

History of psychedelic substances

Psychedelic substances have a rich and fascinating history of use, with evidence suggesting that ancient cultures utilized psilocybin-containing mushrooms for ritualistic purposes as far back as 6,000-7,000 years ago. This practice was particularly notable among the Aztec and Mayan cultures, where the Aztecs referred to these mushrooms as Teonanacatl, meaning “the flesh of God” [19]. In addition, archaeological findings in Texas have revealed the use of mescaline, a 5-HT2A agonist similar to psilocybin, dating back approximately 5,700 years [13].

Various cultures throughout history have employed psychedelics for ceremonial and spiritual purposes. For example, the cult of Demeter in ancient Greece likely engaged in rituals involving psychedelic substances. Similarly, cave paintings in Spain, estimated to be between 6,000 and 8,000 years old, depict a bull alongside psychotropic mushrooms, suggesting their use by ancient tribes [13].

Naturally occurring psychedelics such as psilocybin, mescaline, and salvinorin have been known and utilized for their effects for millennia. While many periods are relevant to their historical use, the most significant milestones for modern research are as follows [20]:

In 1938, Albert Hofmann synthesized LSD at Sandoz Laboratories in Basel, Switzerland. Five years later, in 1943, Hofmann accidentally discovered the psychoactive effects of LSD after ingesting it himself. By 1947, the first clinical trials involving LSD began, and it was marketed as a psychiatric drug until its suspension in 1965.

The 1960s and 1970s saw the commercial availability of psilocybin under the name INDOCYBIN^™^, which was sold in pill form containing 2 mg of psilocybin. However, in 1970, psychedelics were classified as Schedule 1 drugs, effectively halting legal research efforts and severely restricting their study [20].

Research into psychedelics gradually resumed around the year 2000, but progress remains limited due to the regulatory challenges associated with their Schedule 1 status. Notable developments include a 2004 UCLA trial investigating psilocybin for treating pain, anxiety, and depression in cancer patients [20].

Significant breakthroughs in recent years include Compass Pathways Ltd. receiving USFDA approval for “breakthrough therapy” status for psilocybin in the treatment of TRD in 2018, the same year the ketamine analog SPRAVATO^®^ was approved for TRD [20]. In 2019, the Usona Institute also received USFDA “breakthrough therapy” status for a psilocybin treatment targeting MDD [20].

While the cultural use of psychedelics remains alive today, particularly in religious and ritualistic practices, the period from 1938 to 1970 stands out as a crucial era in the scientific exploration of psychedelics. During this time, these substances became popular within the “hippie” culture, but they were also the subject of extensive research before their prohibition.

Despite the outdated methodology of many studies from that period, the sheer volume of research conducted is noteworthy. Over 1,000 clinical papers were published on psychedelics between 1950 and the mid-1960s, involving approximately 40,000 participants [20]. These early studies provided preliminary evidence suggesting that classic psychedelics might be effective in treating conditions such as end-of-life distress and addiction [21].

Proper medical nomenclature of psychedelics and hallucinogens

Since the 1960s, the scientific community has made various attempts to establish a suitable classification and terminology for substances like psilocybin and LSD. Over the years, they have been labeled as entheogens, hallucinogens, psychedelics, and other names.

A more contemporary approach suggests categorizing them as rapid-acting antidepressants due to their swift antidepressant effects [1]. However, this designation remains inadequate, as it fails to account for the consciousness-altering properties of these substances, which are integral to their mechanism of action. This distinction is particularly important as efforts are underway to develop compounds that retain the antidepressant benefits of psilocybin without inducing consciousness-altering or “hallucinogenic” effects.

The therapeutic potential of these substances appears to stem from their neuroplastic and consciousness-altering properties. Based on current literature, it may be more accurate to refer to them as rapid-acting consciousness-altering neuroplastogens. This terminology acknowledges their ability to produce effects similar to SSRIs but within a much shorter time frame [22].

SSRIs are thought to exert their antidepressant effects, at least partially, through neuroplasticity. However, this process typically requires prolonged use over weeks or months. For instance, fluoxetine promotes neuroplasticity by enhancing BDNF/TrkB signaling and inducing BDNF mRNA expression [14]. Additionally, fluoxetine increases extracellular serotonin concentrations through 5-HTT inhibition, but studies indicate that its effects on neuroplasticity markers are independent of 5-HTT inhibition [14].

These findings suggest that depression cannot be solely attributed to low levels of monoamines [1]. Individuals, particularly males, with the Val66Met single nucleotide polymorphism exhibit reduced BDNF release, making them more susceptible to chronic depression [23]. Moreover, they tend to have lower BDNF and TRKB mRNA levels, smaller cortical neurons, reduced synaptic protein levels, and fewer dendritic spines in the prefrontal cortex [23].

The current literature suggests that a more suitable name is needed to emphasize the therapeutic benefits of psychedelics rather than their association with illicit use.

Physical and chemical properties

Psychedelics are typically categorized into three primary groups based on their chemical structure and pharmacological properties [23]. The first group, tryptamines, includes compounds such as DMT and 5-MeO-DMT. The second group, phenethylamines, consists of substances like MDMA, mescaline, DOM, DOI, and others. Finally, the third group, lysergamides, comprises LSD, LDS, and related compounds.

**Figure 1:**
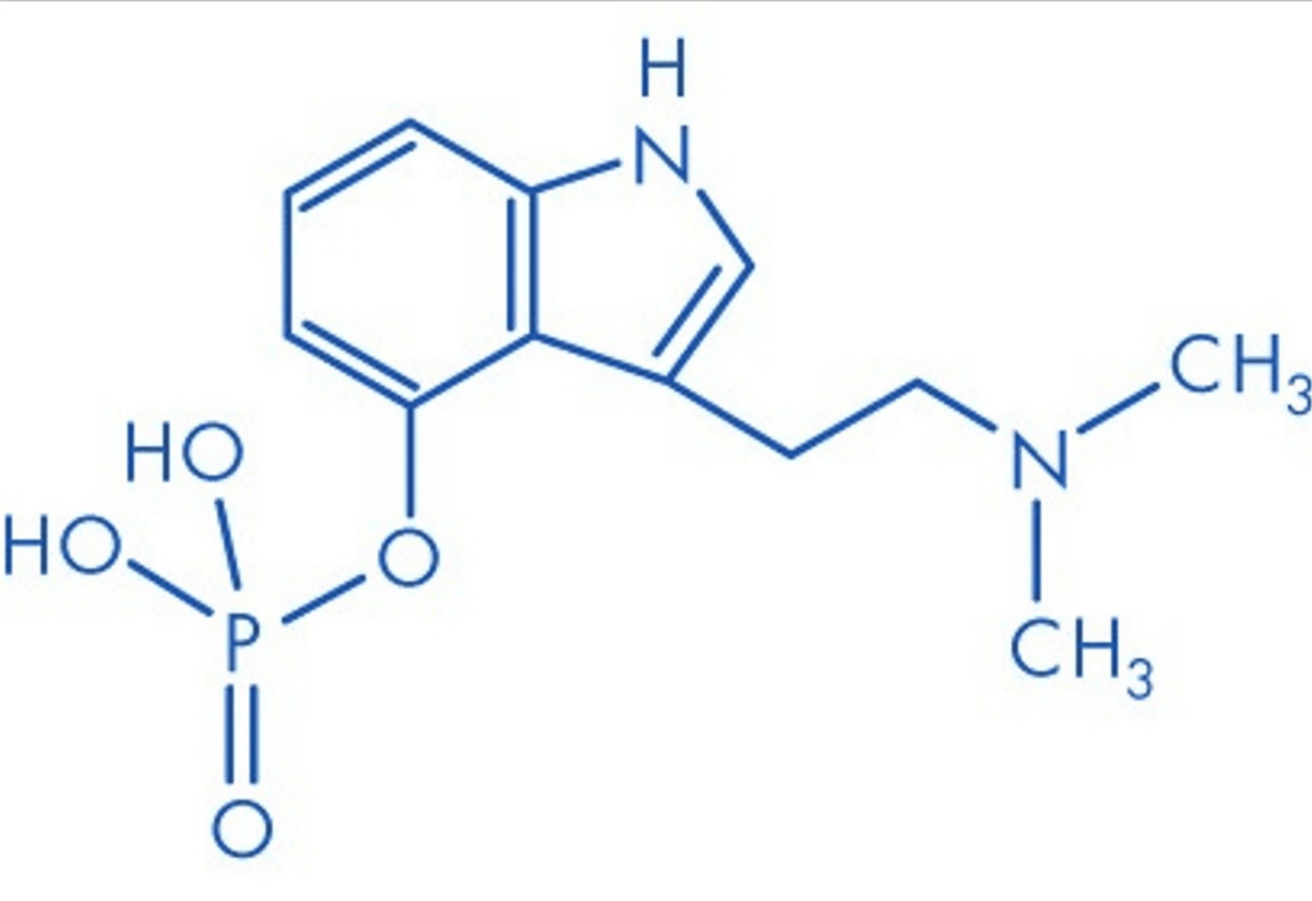
Molecular structure of psilocybin Molecular formula: C12H17N204P; molecular weight: 284.3 g/mol

**Figure 2:**
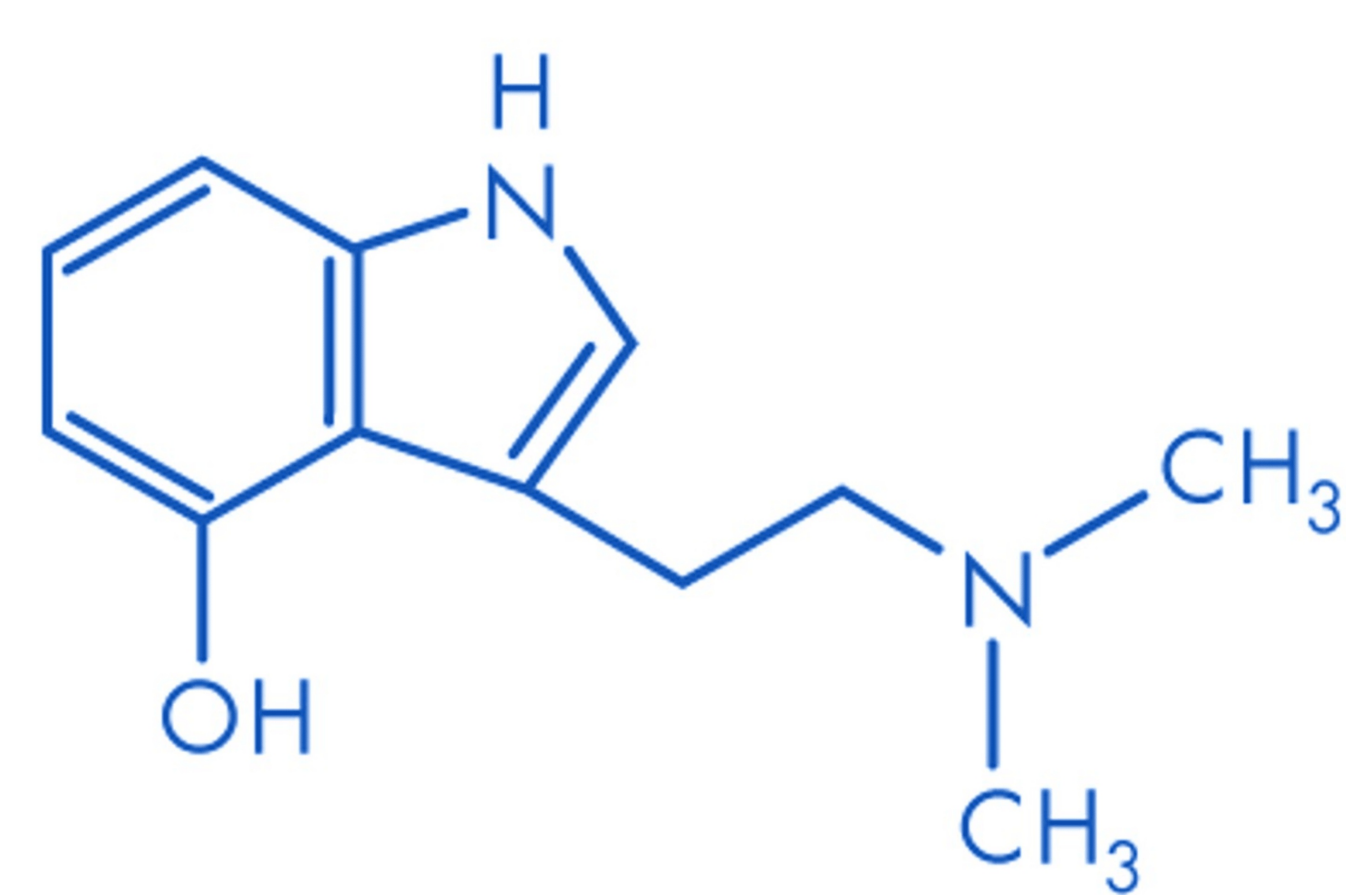
Molecular structure of psilocin Molecular formula: C12H16N20; molecular weight: 204.3 g/mol

Psilocybin [3-(2-dimethylaminoethyl)-1H-indol-4-yl] dihydrogen phosphate and its metabolite psilocin (4-hydroxy-N,N-dimethyltryptamine or 4-OH-DMT) belong to the Tryptamine class of substances, acting as agonists of the 5-HT2A receptor (Table 1). These psychedelics share a structural similarity to serotonin (Figure 1, Figure 2) [24].

**Table 1:** Classification of psychedelics into three biochemical classes: tryptamines, phenethylamines, and lysergamides Psilocybin, psilocin, and mescaline are naturally occurring substances, while the others are synthetic. This list is illustrative and does not include all known substances.

Tryptamines	Phenethylamines	Lysergamines
Psilocybin (3-(2-dimethylaminoethyl)-1H-indol-4-yl)	Mescaline (3,4,5-trimethoxyphenethylamine)	LSD (lysergic acid diethylamide)
Psilocin (4-hydroxy-N,N-dimethyltryptamine)	DOI (2,5-dimethoxy-4-iodoamphetamine)	LSP (lysergic acid 3-pentylamide)
DMT (5-methoxy-N,N-dimethyltryptamine)	DOB (dimethoxybromoamphetamine)	
DET (N,N-diethyltryptamine)		

Psilocybin is soluble in water, methanol, and ethanol, but insoluble in nonpolar organic solvents such as chloroform and petroleum ether [25]. Its melting point ranges from 220 to 228 °C [26]. Notably, two crystalline polymorphs of psilocybin have been identified, along with reported hydrated phases [27].

Psilocin, along with its prodrug psilocybin, forms colorless crystals that are sensitive to temperature. When stored at room temperature for a few months, both substances become deactivated. However, freeze-dried mushrooms can remain active for over two years if stored at -5 °C. The characteristic blue coloration observed in hallucinogenic mushrooms after picking is due to the effects of cytochrome oxidase.

Therapeutic indications

Based on the currently available data, it appears that psilocybin should be approved for two specific indications: (1) the treatment of end-of-life distress and depression in palliative care, whether due to cancer or other underlying causes and (2) the treatment of depression when conventional treatments have not provided clinically significant relief. Several major trials have shown promising results for these indications [24]. While there is also potential for psilocybin to be effective in pain and addiction treatment, these areas fall outside the scope of this review.

Establishing the Therapeutic Dose of Psilocybin

One of the primary challenges in modern psilocybin treatment is the absence of practical guidelines for establishing a standardized therapeutic dose. To effectively treat patients, it is essential to determine an appropriate and consistent dosage. Experimental doses have ranged from 1 mg to over 30 mg, but a systematic review of current literature suggests that a dose of 30 mg per 70 kg provides the most favorable results [28].

A study demonstrated that a single 25 mg dose significantly reduced depression scores over three weeks compared to a 1 mg sub-psychedelic dose, although it was associated with adverse effects such as headache, nausea, and dizziness [1]. Earlier psilocybin trials utilized body weight-based dosing between 0.2 and 0.4 mg/kg, with patients receiving either a single dose or two doses separated by three to four weeks. More recent trials have favored a fixed dose of 25 mg, which has been validated through secondary analyses of previous data [29].

Since the secondary metabolite, psilocin-o-glucuronide, is inactive, patients with mild to moderate renal impairment likely do not require dose adjustments. Fortunately, psilocybin is not considered toxic, with the LD50 for rats determined at 280 mg/kg via intravenous administration. Given that the effective dose is between 25 and 30 mg, reaching a toxic dose of psilocybin in a clinical setting is nearly impossible [5]. The lethal dose in humans is many times higher than the effective dose, making it virtually impossible to consume a fatal amount when using mushrooms or standardized pills.

Mushrooms Versus Pure Psilocybin

Mushroom species that contain psilocybin also possess various other alkaloids besides psilocybin itself. For example, alkaloids such as muscarine, psilocin, aeruginascin, baeocystin, and psilocybin have all been identified in *Inocybe *mushrooms [29].

These compounds may collectively form what is known as a phytocomplex, where the whole plant or mushroom, containing multiple active substances, produces synergistic effects when consumed. Theoretically, achieving the same therapeutic response with a lower dose of psilocybin could be possible if another substance is co-administered. This approach might reduce some of the dose-dependent side effects of psilocybin, although this concept remains purely hypothetical at present.

Using fresh or dried mushrooms in clinical settings is impractical due to the significant variability in the concentrations of psilocybin and other active compounds [19].

It would be valuable to compare the effects of *Psilocybe* genus mushrooms versus pure psilocybin in a clinical setting, as the combined presence of multiple alkaloids may produce somewhat different therapeutic outcomes.

Efficacy

Psilocybin has proven to be an effective treatment option for patients with TRD and for those requiring a rapid therapeutic response, such as terminally ill patients.

Compared to other treatments, psilocybin has shown lasting positive effects, with a ≥50% reduction in the GRID-HAMD score maintained for up to six months after treatment [28,30]. It is likely that studies have predominantly included patients with more severe depression rather than those with mild to moderate symptoms, primarily due to recruitment challenges.

Treatment adherence is a significant issue in psychiatric pharmacotherapy, as patients often struggle to maintain consistent medication use over extended periods. Factors such as forgetting doses, taking incorrect amounts, or timing errors can all contribute to poor adherence. This issue is further compounded when patients are taking multiple medications, which is not uncommon.

Using a single-dose treatment like psilocybin could potentially improve adherence, addressing one of the primary weaknesses associated with standard antidepressant therapies [7].

Psilocybin as a Treatment Option for Depression in Terminal Cancer Patients

Depression is a significant factor in cancer and other severe, debilitating medical conditions [31]. Psilocybin possesses many of the characteristics of an ideal treatment for end-of-life distress. It acts rapidly, produces sustained effects after a single treatment, and carries a low risk of drug-drug interactions due to its one-time dosing.

When patients are experiencing severe suffering or have a limited life expectancy, conventional antidepressants often fail to provide timely and meaningful relief. An epidemiological study involving 10,153 cancer patients in Canada reported that 19% of patients experienced anxiety symptoms, and 12.9% were diagnosed with clinical depression following their cancer diagnosis [31].

To better understand the role of depression in palliative care, public data from Oregon, collected after the implementation of the Death with Dignity Act, offers valuable insight. The majority of patients who received prescriptions for lethal drugs were motivated by nonphysical suffering. While pain or the fear of future pain accounted for only 26.4% of cases, factors like loss of autonomy (91.4%), decreased ability to enjoy life (89.7%), and loss of dignity (77.0%) were the most common reasons for seeking assisted suicide [32].

Psilocybin may be a valuable treatment option when other methods prove inadequate. Studies have shown that, in addition to its antidepressant effects, psilocybin also effectively reduces anxiety [31]. Furthermore, it has been shown to acutely enhance mood in both healthy individuals and patients with cancer or depression [33].

The potential therapeutic benefits of psilocybin for cancer patients include improved adherence to other treatments, reduced depression, decreased end-of-life fear, enhanced quality of life, and a diminished need for additional treatments that may cause drug-drug interactions, such as tricyclic antidepressants (TCAs), SSRIs, and benzodiazepines [31].

Depression significantly impacts the quality of life in cancer patients and other terminally ill individuals. It worsens physical symptoms like pain and fatigue, which can, in turn, reduce adherence to oncologic treatments. Clinically significant depression and anxiety affect approximately 30-40% (or possibly more) of cancer patients receiving hospital care [34].

One study examining psilocybin use in cancer patients found that 60-80% of participants experienced clinically significant reductions in depression and anxiety, along with a more positive outlook on death [34]. Considering that depression is associated with higher mortality, poorer adherence, and subsequently less favorable treatment outcomes [35], psilocybin could be a valuable therapeutic option for oncologic patients, particularly those with terminal illnesses.

Psilocybin in TRD and the “Afterglow Phenomenon”

TRD is a significant medical challenge, particularly due to its prevalence and the frequent failure of psychiatric medications to adequately alleviate symptoms. In some instances, doctors may prescribe four or more medications in an attempt to achieve even partial therapeutic relief [6]. The primary goal is to reduce depressive symptoms and facilitate the initiation of effective psychotherapy. However, patients experiencing high stress levels or severe depressive states are far less likely to respond positively to psychotherapy and counseling.

After psilocybin administration, a phenomenon known in the literature as the “afterglow phenomenon” often occurs. This refers to a pattern of subacute effects that persist well beyond the drug’s elimination from the body [36]. During this phase, patients are notably more receptive to psychotherapeutic interventions, making it essential to initiate intensive psychotherapy within 15 days following psilocybin treatment.

Psilocybin could serve as a powerful tool for addressing TRD, particularly when standard treatments fail to produce meaningful results. It should be considered a viable option for patients with TRD who do not have psychotic comorbidities. Furthermore, it offers a more humane alternative compared to electroconvulsive therapy.

Safety profile of psilocybin

The safety profile of psilocybin is often misrepresented, with some viewing it as an exceptionally safe substance and others as a highly dangerous drug with no therapeutic value. Neither of these perspectives is accurate. Psilocybin should be regarded as a medical drug, possessing both therapeutic and adverse effects, much like any other medication currently on the market. Compared to similar substances, psilocybin is generally considered to have the most favorable overall safety profile [37].

One of the key advantages of psilocybin over conventional antidepressants is that treatment typically involves a single dose. As a result, most adverse effects occur during or shortly after the treatment session, whereas adverse effects from SSRIs can be prolonged depending on the duration of treatment. Common antidepressants are associated with long-lasting adverse effects such as weight gain, sexual dysfunction, insomnia, sleepiness, and a high potential for drug interactions.

The somatic adverse effects of psilocybin include transient increases in blood pressure, tachycardia, headaches, and gastrointestinal distress [7,38].

One study reported a moderate elevation of systolic blood pressure following psilocybin administration, with no significant changes in serum cholesterol, alkaline phosphatase, cholinesterase, or aspartate aminotransferase levels two hours post-administration. Mild increases in body temperature were also observed. The authors concluded that there were no concerns regarding potential somatic health [7,38]. Physiological side effects caused by psychedelics are generally moderate at best [9].

Psilocybin is considered safe when used in a controlled and supervised environment. According to the UK Independent Scientific Committee on Drugs’ 2010 report, psilocybin mushrooms were regarded as the least harmful among the controlled substances in the UK, with drug-related impairment posing far less danger compared to alcohol, benzodiazepines, and even tobacco [5].

Regarding toxicity, an older study determined the LD50 of intravenous psilocybin to be above 250 mg/kg, with 200 mg/kg causing no fatalities and 250 mg/kg resulting in the death of a small portion of animals [39]. More recent findings established the LD50 at 280 mg/kg via intravenous infusion [5].

There is one rare documented case of fatal psilocybin overdose involving a 24-year-old female who had undergone a heart transplant 10 years earlier due to end-stage rheumatic heart disease. The patient suffered cardiac arrest two to three hours after consuming mushrooms containing psilocybin and subsequently died. Autopsy results detected only THC and psilocin, the active metabolite of psilocybin [39].

Adverse psychiatric events are more commonly associated with high doses of psilocybin (>25 mg), with the most frequently reported issues including transient anxiety, paranoia, delusions, panic attacks, derealization, depersonalization, confusion, and dissociation [7,40,41]. Even with smaller doses, psychiatric and physical adverse reactions have been observed in approximately 20% of cases [1]. However, severe psychiatric effects are rare in clinical settings, and the risk of prolonged psychosis lasting more than two days is extremely low.

Psilocybin does not cause dependence since it does not directly affect the dopaminergic system, nor does it impair liver function or cause tissue toxicity [5]. Notably, psilocybin induces immediate tolerance, meaning that taking consecutive doses produces minimal effects [6,7]. There is no evidence suggesting that psilocybin causes withdrawal symptoms [7].

Additionally, another study found no evidence that psilocybin negatively affects memory consolidation [42]. Population studies have shown no association between lifetime psychedelic use and mental illness, suicide, or suicide attempts. In fact, they suggest the opposite, indicating that psychedelic use may lower the need for mental health treatment compared to the general population [9,41].

Proper preparation before treatment is of utmost importance to minimize the risk of adverse psychiatric effects, commonly referred to as a “bad trip.” Most psychiatric adverse effects can be avoided with adequate preparation. The patient should be relaxed and free from any other substances, with counseling provided beforehand by a trained psychiatrist [6].

One very rare but potentially serious adverse reaction to psilocybin is hallucinogen persisting perception disorder (HPPD) [2]. HPPD is a condition where patients continue to experience lingering psychedelic effects long after the substance has been metabolized and eliminated from the body. Unfortunately, this condition is poorly understood, and treatment options are limited. HPPD is most commonly observed in individuals who have taken LSD, and its incidence following psychedelic use is estimated to be only a few cases per million users [40]. It is often refractory to treatment, with lamotrigine considered the drug of choice [43].

There is currently a lack of information on the effects of psilocybin in patients with psychotic disorders or those with a family history of such disorders. These patients are likely to benefit less from psilocybin treatment compared to those with unipolar depression. Clinical trials consistently exclude individuals with psychotic disorders [19].

Psychotic disorders, including schizophrenia, are generally considered absolute contraindications for psilocybin use. Reports of auto-mutilation and self-destructive behavior have been documented in individuals consuming mushrooms containing psilocybin [32]. Research conducted during the pre-prohibition era demonstrated that patients with psychotic disorders, such as mania and schizophrenia, often experienced worsening symptoms following psychedelic treatment [9]. This risk is why individuals with bipolar disorder and psychosis are consistently excluded from clinical trials [24,44]. Importantly, no prolonged psychotic-like reactions have been reported in other patient populations within clinical trials over the past 30 years [9,44].

Psilocybin is currently classified as a Schedule I substance, which indicates a high potential for abuse, lack of approved therapeutic use, and an inability to be safely used in medicine. Its classification can only be changed if it receives approval for therapeutic use. The appropriate scheduling of psilocybin would be determined by evaluating the eight factors outlined in the Controlled Substances Act (CSA) [39].

One study found that psilocybin has a lower risk of dependence than caffeine and is among the least psychologically toxic substances, with minimal risk of death [39]. By comparison, nicotine meets the criteria for a Schedule III drug. Considering psilocybin’s favorable safety profile and the CSA’s eight factors, it is likely to be classified as a Schedule IV drug if approved for medical use [39].

Most clinical trials indicate that psilocybin provides fast-acting, beneficial, and lasting therapeutic effects with a generally good safety profile. Adverse effects are usually mild and temporary, commonly including nausea, anxiety, confusion, vomiting, and headaches [41].

Although more research is needed, early findings suggest that psilocybin has a favorable safety profile when used appropriately. While psilocybin offers promising therapeutic benefits, it still carries some potential for abuse and associated risks. Additionally, because there is no data on the effects of psilocybin on fetuses, pregnant and breastfeeding women should avoid using psilocybin.

Drug Interactions With Psilocybin

The lack of data on drug-drug interactions involving psilocybin is primarily due to the fact that most studies require participants to discontinue other medications before treatment. This information gap is critical, as most patients eligible for psilocybin therapy are likely to be on one or more medications, such as benzodiazepines, lithium, SSRIs, monoamine oxidase inhibitors (MAOIs), opioids, painkillers, and similar drugs.

A theoretical concern is the risk of serotonin syndrome when psilocybin is combined with SSRIs. Additionally, prolonged SSRI use may reduce psilocybin’s effectiveness due to 5-HT2A receptor downregulation [2,24]. However, a study involving a two-week pretreatment with escitalopram found no significant reduction in psilocybin’s efficacy and even noted a decrease in adverse effects. While this suggests that escitalopram and possibly other SSRIs may be safely combined with psilocybin, the study’s small sample size means more research is necessary before drawing firm conclusions [45].

Antipsychotics and other 5-HT2A antagonists, such as quetiapine and olanzapine, are known to block psilocybin’s effects and should not be administered alongside it [46].

Lithium is considered absolutely contraindicated when used with psilocybin. In a study examining 62 reports of lithium use with psychedelics (LSD or psilocybin), seizures occurred in 47% of cases, while “bad trips” were reported in 18% of cases. Comparatively, reports involving lamotrigine with psychedelics (LSD, psilocybin, or DMT) showed no seizures or “bad trips,” with the psychedelic experience being unaffected in 65% of lamotrigine cases versus only 8% of lithium cases [47].

MAOIs can amplify psilocybin’s psychedelic effects, so their use should be discontinued before psilocybin treatment [48]. Caffeine may enhance stimulation and further elevate blood pressure, while tramadol poses a serious interaction risk by lowering the seizure threshold [48]. Substances such as ethanol, gamma hydroxybutyric acid, and benzodiazepines can diminish psilocybin’s effects, while cannabis may cause either relaxation or severe anxiety [48]. Amphetamines can increase the risk of experiencing a “thought loop,” a condition where the user feels trapped in a repetitive sequence of thoughts or ideas [48].

Although food interactions are not well documented, one potential concern is St. John’s Wort, as it acts similarly to SSRIs and could interact with psilocybin.

Mechanism of action

The exact mechanism through which psilocybin exerts its effects is not fully understood, but this is also true for many common antidepressants and other CNS drugs. However, there is growing evidence suggesting that all antidepressant agents, including psilocybin, induce neuroplastic changes in the brain, thereby modifying dysfunctional neural pathways.

Neuroplasticity refers to the brain’s ability to reorganize itself by forming new neural connections or altering existing ones. One relevant theory is Hebbian plasticity, which describes the principle that neurons that frequently interact undergo changes to enhance their ability to communicate, essentially “wiring together” through repeated activation [49].

Psilocybin primarily interacts with serotonergic neurotransmission, particularly through the 5-HT1A, 5-HT2A, and 5-HT2C receptor subtypes. The equilibrium dissociation constants (Ki) are approximately 6 nM for the 5-HT2A receptor and 190 nM for the 5-HT1A receptor [38]. In addition to its serotonergic effects, psilocybin exhibits minor activity on adrenergic, dopaminergic, and histaminergic systems [9].

One of psilocybin’s key mechanisms involves the downregulation of 5-HT2A receptors, which are often overexpressed in patients with MDD. This downregulation may be influenced by increased BDNF synthesis in the medial prefrontal cortex (mPFC). Activation of the 5-HT2A receptor has a positive effect on cerebral neuroplasticity [2].

The prevailing hypothesis suggests that depression is not primarily caused by a deficiency in monoamines but rather by inadequate or altered production and maturation of neurons. Reduced volume of the mPFC and hippocampus is one of the most frequently observed abnormalities in patients with depressive disorders.

Proteins that regulate cell proliferation, plasticity, and neural function maintenance are known as neurotrophins, which include NGF, NT-3, and NT-4. Proneurotrophins interact with the p75NTR receptor to mediate neuronal death, reducing plasticity. In contrast, mature neurotrophins bind to tyrosine kinase receptors (Trk), promoting differentiation and survival through enhanced branching of both axons and dendrites [30].

The neurotrophic hypothesis is further supported by postmortem studies showing reduced BDNF levels in the cerebral cortex of individuals who died by suicide. Increased levels of mature BDNF through TrkB signaling are believed to produce an antidepressant response, while pro-BDNF interacting with p75NTR signaling may have a depressive effect [1]. Notably, several antidepressants, including SSRIs and ketamine, can also directly bind to the TrkB receptor [16].

Furthermore, activation of 5-HT2A receptors increases the excitability of the visual cortex even in the absence of external visual stimuli [50]. This receptor activation may also enhance dopamine release from the striatum, potentially regulating the defective reward pathway often seen in depressed patients.

Psilocybin-induced BDNF activation can also stimulate AMPA and NMDA receptors, promoting downstream effects like gene expression, signal transduction, enhanced plasticity, and neurogenesis [13].

Psilocybin increases sensory and brain-wide connectivity while reducing associative connectivity [51]. Additionally, it has been shown to normalize amygdala hyperactivity and alleviate negative moods associated with depression [6]. Though psilocybin is believed to affect the default mode network (DMN), some studies suggest that changes observed in the DMN are not exclusive to classic psychedelics and may occur with other mind-altering substances as well [2].

The DMN plays a critical role in consciousness, self-construction, and cognitive integration. One fMRI study demonstrated that psilocybin reduces activity and connectivity in the brain's connector hubs, resulting in a more unconstrained style of cognition [52].

An additional mechanism of action involves the reduction of pro-inflammatory cytokines, such as TNF-α and IL-1β. Elevated levels of these cytokines can activate the kynurenine pathway within the microglia, leading to the production of neurotoxic compounds like hydroxykynurenine and quinolinic acid [1]. These compounds contribute to neurotoxicity through the generation of reactive oxygen species that can damage neural cells and act as agonists at NMDA receptors.

Kynurenine metabolism varies depending on the cell type involved in production, metabolism, and transport. One meta-analysis noted decreased levels of kynurenine and kynurenic acid in depressed patients, while higher levels of quinolinic acid were found in non-depressed individuals [1]. Factors such as hyperactivation of the hypothalamic-pituitary-adrenal axis, chronic low-level inflammation, and neuroplasticity dysregulation are believed to play roles in the etiology of depressive disorders [9,14].

Evidence suggests that psilocybin reduces blood flow to the amygdala within one day of treatment, with this reduction correlating with decreased depressive symptoms [30].

There is much debate about whether the psychedelic experience itself is necessary for therapeutic benefits. Ideally, non-psychedelic compounds producing the same beneficial effects as psilocybin would be preferred. However, the psychedelic experience may be an integral part of the treatment process, as spiritual or mystical experiences have been associated with sustained psychological benefits [2,7]. While drug dosage is a crucial determinant of treatment response, the therapeutic effects are also influenced by the environment in which the patient is treated and individual characteristics [53].

Remarkably, psilocybin and other psychedelics produce lasting therapeutic effects days after administration, despite being eliminated from the body. This suggests that the positive outcomes are likely due to persistent changes in brain function resulting from their use [9].

It has been established that the potency of classical psychedelics like psilocybin and LSD depends on their ability to bind to 5-HT2 receptors [48].

**Figure 3:**
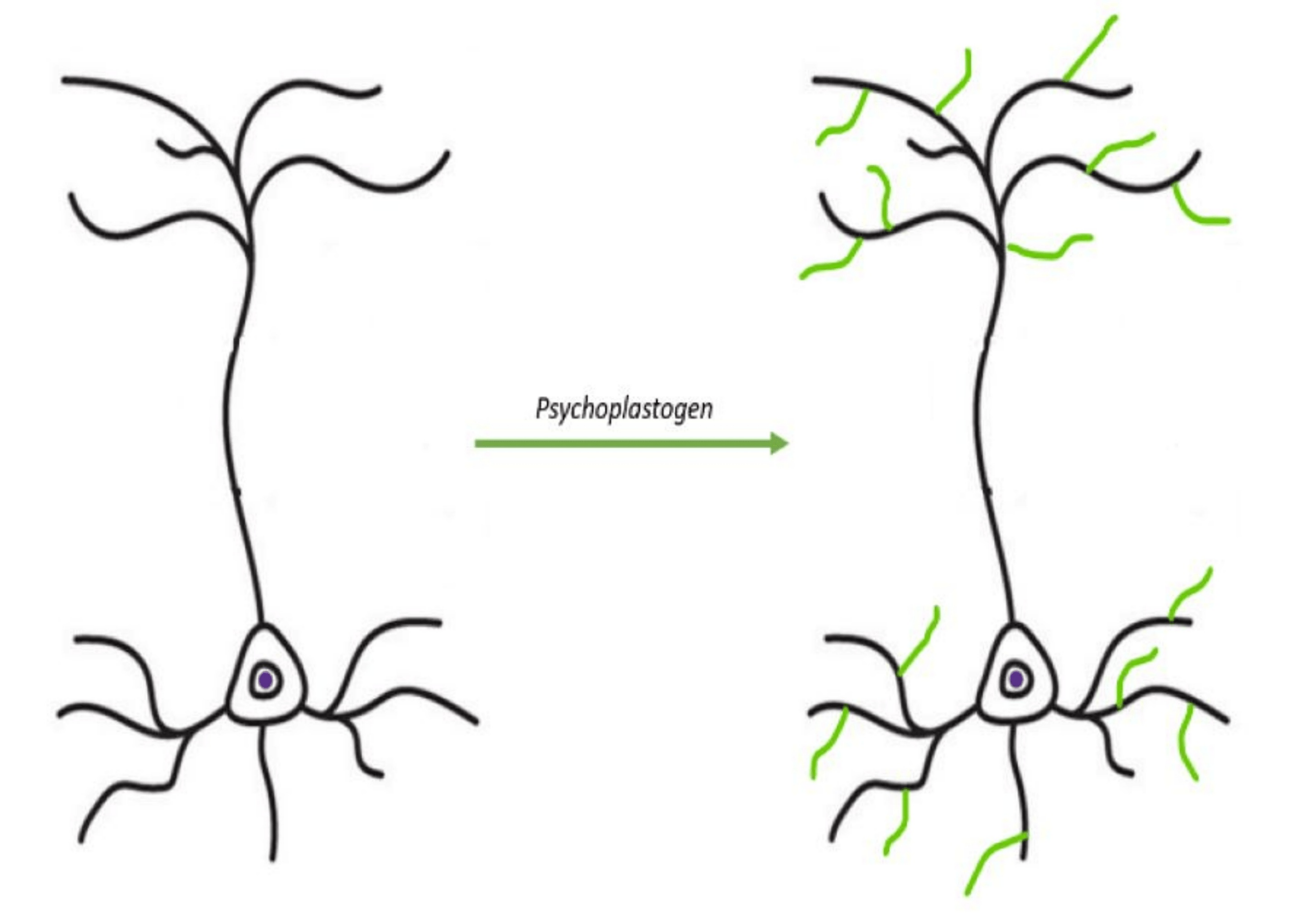
Cortical atrophy and reversal by psychoplastogens Cortical atrophy observed in depression is characterized by the retention of dendritic branches and the loss of spines. Psychoplastogens promote neural regeneration and connectivity, effectively reversing these structural changes.

**Figure 4:**
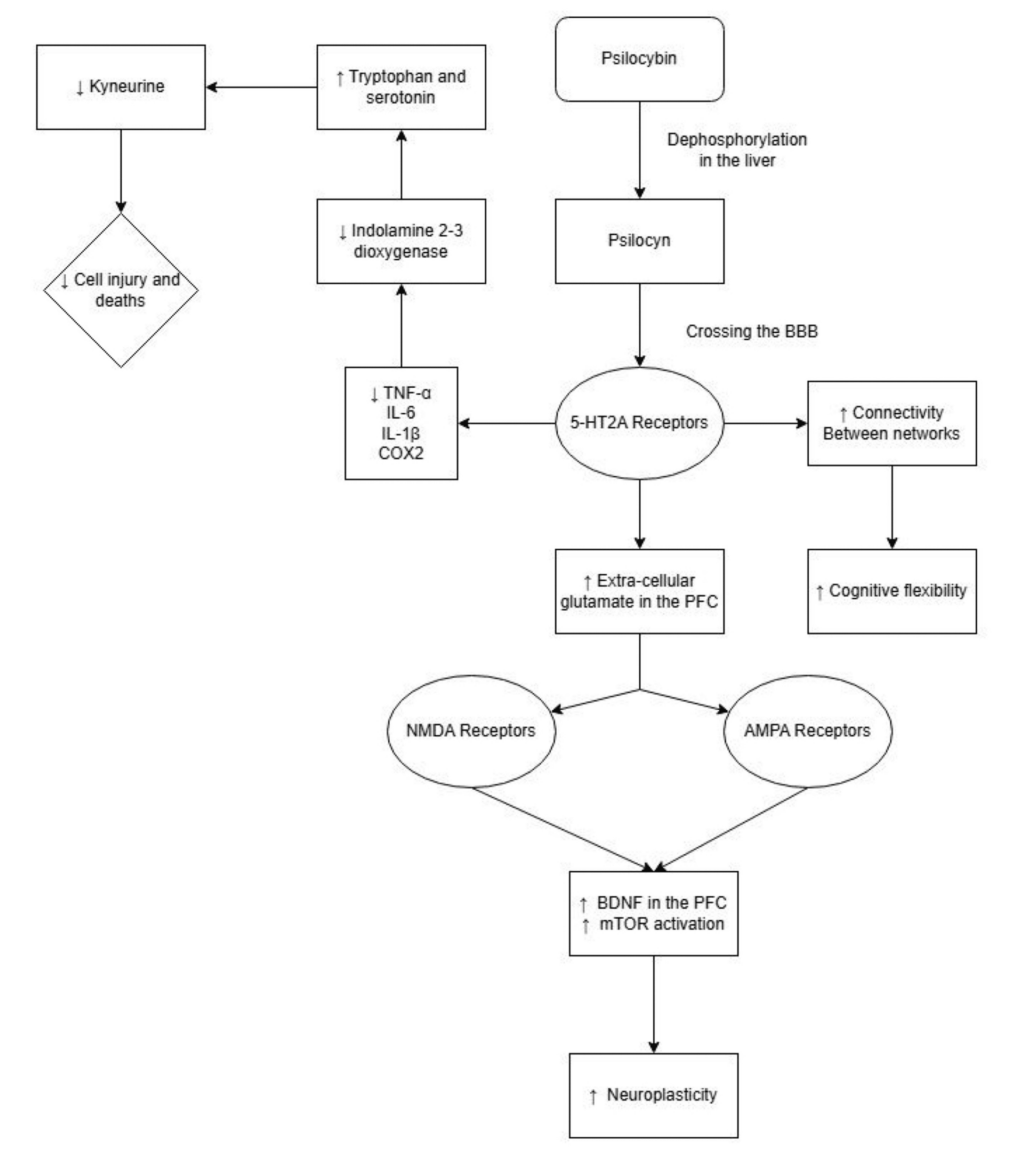
Schematic representation of psilocybin’s mechanism of action

Literature findings suggest that, in simplified terms, psilocybin’s mechanism of action essentially “presses a deep-rooted reset button,” allowing the CNS to effectively heal and rewire itself through neuroplastic mechanisms (Figure 3). Current literature provides a detailed graphical representation of this mechanism of action (Figure 4) [48].

Pharmacokinetics

The effects of psilocybin typically begin within 10-40 minutes after ingestion and can last anywhere from two to six hours, depending on the dose [2,54]. Psilocybin undergoes first-pass metabolism, resulting in a significant reduction in its concentration before it reaches systemic circulation. It is primarily metabolized in the liver and intestines, where it is converted to psilocin, the active form of the drug. Psilocin is then extensively distributed through the bloodstream to all tissues, displaying linear pharmacokinetics.

Recent studies using a fixed 25 mg dose of psilocybin and specifically measuring unconjugated psilocin have reported a half-life (T1/2) of approximately 108 minutes, with a range of 66-132 minutes. After this fixed oral dose, one study noted a maximum psilocin plasma concentration (Cmax) of 20 ng/mL at 120 minutes (Tmax), while another reported a Cmax of 18.7 ng/mL [24].

Psilocin is produced through the dephosphorylation of psilocybin in the intestinal mucosa [55]. Unlike psilocin, psilocybin likely cannot freely cross the blood-brain barrier due to its lower lipophilicity [56]. The bioavailability of psilocybin after oral ingestion is approximately 50% [24].

Psilocybin is eliminated primarily through the kidneys, with two-thirds of the excretion occurring within three hours of ingestion [30]. Psilocin and its metabolites are mostly excreted in the urine (65% of the dose within 24 hours) with bile elimination accounting for 15-20%. While most metabolites are cleared within eight hours, up to 20% may be retained for longer periods, with significant quantities detectable in urine up to seven days post-administration [56].

An open-label study was conducted to assess the pharmacokinetics and, to some extent, the safety profile of psilocybin. This study administered escalating oral doses of 0.3, 0.45, and 0.6 mg/kg to 12 healthy adults approximately once a month in a controlled setting, with subjects monitored for 24 hours [57]. Blood and urine samples were collected and analyzed using a validated liquid chromatography-tandem mass spectrometry assay to detect psilocybin and psilocin.

Psilocybin was not detected in plasma or urine, and renal clearance of psilocin accounted for less than 2% of total clearance. The pharmacokinetics were linear, with an elimination half-life of approximately three hours. An extended elimination phase observed in some subjects suggests hydrolysis of the psilocin glucuronide metabolite. Two studies confirmed that psilocybin is rapidly converted to psilocin after oral ingestion, with psilocin having a half-life of 163 minutes and predominantly found in the glucuronide form (67%) [57]. Psilocin-O-glucuronide is the primary urinary metabolite with clinical and diagnostic relevance.

The remaining 20% of absorbed psilocin undergoes oxidation, often involving demethylation and deamination to produce 4-hydroxyindole-3-acetaldehyde, 4-hydroxyindole-3-acetic acid, or 4-hydroxytryptophol [48]. Following oral administration, plasma concentration peaks occur at 80-105 minutes post-ingestion. In contrast, intravenous administration of psilocybin results in faster mean maximum plasma level peaks of psilocin, recorded at 12.9 ± 5.6 ng/mL approximately 1.9 ± 1.0 minutes post-injection [56].

Since psilocybin itself is undetectable in plasma or urine, it is evident that it is rapidly dephosphorylated into psilocin upon ingestion. Metabolites such as psilocin-O-glucuronide and 4-hydroxyindole alcohol are present in concentrations several-fold higher than psilocin in plasma. The renal clearance of psilocin is estimated to be between 2% and 3.5% of total clearance [57]. Notably, no serious adverse effects were reported during the study [57].

The economics of psilocybin

While patient health and well-being remain the top priority, the economic aspect of treatment cannot be overlooked. Financial considerations should be seen not only as monetary costs but also as resource consumption. Ideally, effective treatments would not demand specialized personnel or complex machinery. Although psilocybin therapy requires trained psychiatrists and carefully designed environments with calming, therapeutic settings, it has the potential to be a cost-effective option.

An essential aspect of this discussion is the production of psilocybin itself. There are several methods to obtain psilocybin, each with its own economic implications:

Extraction From Mushrooms

**Figure 5:**
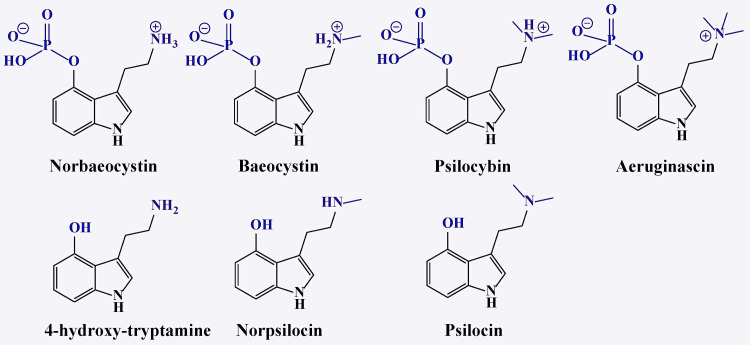
Tryptamines present in Psilocybe mushrooms

Approximately 80 different species of *Psilocybe* mushrooms contain psilocybin along with other related compounds, such as norpsilocin and aeruginascin (Figure 5). However, extracting psilocybin from these mushrooms is a complex process, as the drying procedure can significantly affect dephosphorylation [58].

Producing Synthetics Psilocybin

There are several methods for synthesizing psilocybin on an industrial scale. One effective approach involves the Speeter-Anthony tryptamine pathway, which can produce psilocybin with 99.9% purity. Cost-efficient methods exist for synthesizing psilocybin, although psilocin, the active form, remains unstable in solution. Additionally, it is feasible, and potentially simpler, to synthesize other prodrugs such as psilacetin [58].

Biosynthesis

Mass production of psilocybin and similar compounds through biosynthesis is theoretically possible using genetically modified microorganisms, employing technology similar to that used for producing insulin or antibiotics. This approach holds significant potential for future production. For instance, genetically modified *Escherichia coli *has achieved a production level of 1.16 g/L, although the high cost of the substrates used remains a notable drawback [1].

Considering the standard psilocybin dose of 30 mg per 70 kg, producing adequate quantities should not pose a significant challenge, as one kilogram of psilocybin could potentially treat approximately 30,000 patients.

However, the primary drawback in terms of resource utilization is the necessity of having a trained psychiatrist present during treatment. This challenge is further compounded by the duration of the treatment, which often lasts three hours or more, with some sessions extending to six to eight hours [59]. Consequently, psilocybin therapy may not be suitable as a first-line treatment for every patient (Table 2).

**Table 2:** Comparison of psilocybin versus standard treatments ^*^ Includes the price of the drug, hospital resources, patient work performance, quality of life, use of sick days, and other related factors MAOI, monoamine oxidase inhibitor; SSRI, selective serotonin reuptake inhibitor

Aspect	Psilocybin	Standard treatments (SSRIs or MAOIs)
Dosing	A single dose is required to achieve treatment response.	Long-term use is required.
Effect onset	Immediate	Takes three weeks or more
Adverse effects	Mild based on current data but insufficiently documented	Common, ranging from mild to severe and life-threatening, but well documented
Adherence	Ideal, as treatment is administered in-house with a single dose	Poor, as patients often skip or forget to take medication
Resource utilization^*^	More resource-intensive short term, but less intensive long term	Less resource-intensive short term, but potentially more intensive long term
Drug interactions	Poorly documented; less likely to occur due to single dosage	Well documented; more likely to occur due to prolonged use

Conducting proper studies on psilocybin: challenges with microdoses as active placebos and blinding

Studying the therapeutic effects of psilocybin in a clinical setting presents significant challenges. Implementing single or double-blind protocols is technically impossible because the effects of psilocybin and other psychedelics are readily apparent to both the patient and the therapist, making true blinding infeasible [6,60].

One alternative approach involves using a small, sub-psychedelic dose of psilocybin as a comparator, as applied in some studies. However, this method is also problematic since the effects of low doses of psilocybin are not yet fully understood. There is a growing trend of using small, non-psychedelic doses of psilocybin and other substances, commonly referred to as microdosing. Microdosing generally involves taking a small amount of a psychedelic, typically 5-10% of a full dose, which does not produce psychedelic effects [61]. The Fadiman protocol is a well-known regimen suggesting a dose every third day.

Although most studies have not demonstrated particular therapeutic benefits from microdosing, it is possible that these studies were not conducted for long enough to detect meaningful changes [60,61]. Despite the lack of significant findings indicating that microdosing has antidepressant effects, it remains possible that a longer duration of use - similar to the time required for common antidepressants to show efficacy - may be necessary to produce therapeutic effects. This could be related to the time needed to achieve neuroplastic changes. Therefore, using a smaller dose is problematic, as it may have therapeutic effects over the long term [60]. Some studies have also employed niacin as an active placebo [5].

Another potential solution is to use standard antidepressants, such as fluoxetine or sertraline, as comparators. Notably, a study by Simonsson et al. compared the effects of psilocybin to escitalopram and found that psilocybin treatment demonstrated comparable efficacy to escitalopram, with higher response and remission rates [17].

Ideally, a well-designed study would include a large group of patients receiving psilocybin treatment alongside two control groups receiving approved antidepressants. While it is possible to include a placebo group (receiving a placebo instead of fluoxetine or sertraline, not psilocybin), ethical concerns arise when approved treatments are available. Given that psilocybin is a promising candidate for treating end-of-life distress and TRD, the use of a placebo in such cases is ethically questionable.

Another significant limitation of current studies is the small number of participants; many studies involve only 10-20 patients. The largest trial to date was conducted by the Goodwin team, involving 233 participants from Europe and North America divided into three groups receiving a single dose of psilocybin (1 mg control, 10 mg, and 25 mg) [59].

To obtain more reliable results, studies need to include larger and more diverse participant groups. Ideally, these participants should be psychedelic-naive, meaning they have never used any psychedelic substances before. Additionally, participants should represent diverse backgrounds and include both male and female patients, those with comorbidities, and individuals from various ethnicities. Notably, in most studies (70.6%), approximately 75% of participants were white [62].

Proposed treatment guideline

Based on the currently available data, the following guideline is proposed for treating patients with psilocybin, should it receive proper regulatory approval:

Preparatory Stage

Before initiating treatment, several steps should be followed to ensure patient safety and optimize therapeutic outcomes. If the patient is taking any contraindicated or potentially contraindicated medications or illicit substances, a washout period of at least seven days should be implemented, or longer if clinically indicated based on the pharmacokinetics of the substances involved. If there is a risk of symptom worsening, a tapering regimen should be employed before discontinuation.

In cases where there is a risk of self-harm, the patient must be hospitalized prior to beginning the washout process. During the examination, it is essential to verify the absence of contraindicated somatic conditions, such as significant cardiovascular diseases (e.g., previous heart transplant, stroke, or heart failure), severe hypertension, or epilepsy. Additionally, any conditions that could potentially affect treatment, such as a history of severe allergic reactions, should be documented.

It is crucial to confirm that the patient does not have a psychotic condition, including but not limited to bipolar disorder or schizophrenia. Before treatment, a standard questionnaire (such as the Beck Depression Inventory) should be completed.

Once the patient has undergone medical and psychiatric evaluation and, if necessary, a washout period, they should be hospitalized 24 hours before treatment. Counseling should be provided during this period, with the patient encouraged to be relaxed the day before treatment. During counseling, the patient needs to be informed about the psychedelic experience and what it might entail. Simulating the experience using a virtual reality set is recommended.

Furthermore, the patient should have a minimum of eight hours of sleep before the treatment session to ensure they are well rested.

Drug Administration Stage

Before beginning treatment, ensure that ketanserin or a similar medication such as risperidone is readily available to address potential severe adverse reactions, such as violent hallucinations, that may require immediate cessation of the treatment. Allow the patient to relax and become accustomed to the environment, which should be a well-lit room with a calm and soothing atmosphere.

Administer an oral dose of psilocybin at 30 mg per 70 kg of body weight. Continuously monitor the patient’s vital signs throughout the session, with at least one qualified staff member present at all times to provide reassurance if needed.

Post-Administration Stage

Once the patient has regained full consciousness, allow them to relax for an additional 30 minutes before assessing treatment efficacy using the same standardized questionnaire used during the preparatory stage. Following this assessment, initiate the first psychotherapy session.

If there are no associated post-treatment risks, such as elevated suicide risk, the patient can be discharged, with explicit instructions not to drive or consume any other substances for the remainder of the day.

Over the next 15 days, conduct regular psychotherapy sessions at a minimum frequency of once every five days, increasing the frequency if deemed necessary or beneficial. After completing the initial set of sessions, continue with psychotherapy sessions every three months for the first year.

## Conclusions

Psilocybin shows potential as a treatment for depressive disorders, particularly for cases of TRD and depression in terminally ill patients. While SSRIs, TCAs, and MAOIs will likely remain the primary and secondary treatment options until further clinical trials establish psilocybin's efficacy and safety, the ability of psilocybin to produce rapid improvement with a single dose and sustain a prolonged therapeutic response is a significant advantage. While more research is certainly needed, regulatory approval may be considered by relevant authorities for use in the aforementioned cases. It is important to emphasize that psilocybin is best used in controlled clinical environments. Making it easily accessible to the general public or allowing untrained personnel to administer it would pose serious risks, including accidental injury or misuse. Further research should focus on evaluating the long-term effects of psilocybin, particularly the duration of positive outcomes and whether psychotherapy enhances treatment efficacy after drug administration. Additionally, it is crucial to investigate potential interactions between psilocybin and commonly prescribed medications, such as SSRIs, to identify any significant effects. Finally, research must also determine whether the psychedelic experience itself is essential for achieving therapeutic benefits.
